# Comparative Evaluation of Systemic Inflammatory Indices in Bronchiectasis: Identification of Exacerbation Phenotype

**DOI:** 10.3390/life16060949

**Published:** 2026-06-04

**Authors:** Selda Günaydın, Hayriye Bektaş Aksoy, Şaban Melih Şimşek

**Affiliations:** Department of Chest Diseases, Giresun University Faculty of Medicine, Giresun 28100, Türkiye; bektas_hayriye@hotmail.com (H.B.A.); melih.simsek@giresun.edu.tr (Ş.M.Ş.)

**Keywords:** bronchiectasis, inflammation, exacerbation, biomarkers

## Abstract

Background: Bronchiectasis is a heterogeneous chronic inflammatory airway disease characterized by recurrent exacerbations. Data on composite inflammatory biomarkers for assessing disease activity remain limited. Methods: This retrospective study included 97 patients with non-cystic fibrosis bronchiectasis categorized as stable (n = 39) or with exacerbated bronchiectasis (n = 58). Demographic, clinical, and laboratory data were analyzed, and inflammatory indices—NLR (neutrophil–lymphocyte ratio), PLR (platelet–lymphocyte ratio), SII (Systemic Immune-Inflammation Index), PIV (Pan-Immune-Inflammation Value), CAR (C-reactive protein-to-albumin ratio), and HALP score (hemoglobin × albumin × lymphocyte/platelet)—were calculated, followed by multivariate logistic regression and ROC analyses. Results: Patients with bronchiectasis exacerbations had a higher NLR, PLR, PIV, SII, and CAR and lower HALP (all *p* < 0.001). The C-reactive protein-to-albumin ratio demonstrated the highest discriminative ability (AUC = 0.995), followed by SII and NLR, while lower HALP and SII were independent predictors of exacerbation. The C-reactive protein-to-albumin and sedimentation-to-albumin ratios were strongly correlated with hospitalization duration (both *p* < 0.001). Conclusions: Composite inflammatory indices are strongly associated with disease activity in bronchiectasis. CAR showed excellent discriminative performance, while HALP and SII independently predicted exacerbation. These simple, cost-effective biomarkers may support risk stratification and clinical monitoring in routine practice.

## 1. Introduction

Bronchiectasis is a chronic, heterogeneous respiratory disease characterized by irreversible bronchial dilatation, persistent airway inflammation, recurrent infections, and progressive structural lung damage [1,2,3,4]. The clinical course of the disease is highly variable, with some patients remaining stable while others experience frequent exacerbations associated with increased morbidity, hospitalization, and mortality, particularly in exacerbation-prone patients [5].

The pathophysiology of bronchiectasis arises from a complex interaction between impaired mucociliary clearance, chronic infection, and persistent inflammation, often described as a “vicious cycle” that sustains airway damage [5,6]. Among these mechanisms, neutrophil-dominant inflammation plays a central role, contributing to airway destruction, protease imbalance, and disease progression [5]. Bronchiectasis involves both airway and systemic inflammation, with the latter increasing during exacerbations and persisting even after clinical improvement, highlighting the need for reliable biomarkers reflecting systemic inflammatory burden [7].

Due to the heterogeneous nature of bronchiectasis, there is increasing interest in identifying reliable, readily available biomarkers that can reflect disease activity, predict exacerbations, particularly in exacerbation-prone patients, and guide clinical management [2]. Traditional inflammation markers such as C-reactive protein (CRP) and the erythrocyte sedimentation rate (ESR) are widely used; however, these markers alone may not fully reflect the complex and multi-dimensional status of inflammatory processes in chronic respiratory diseases [8].

In recent years, complex inflammatory markers derived from routine hematological parameters have been suggested as promising tools for assessing systemic inflammation. Among these, the neutrophil-to-lymphocyte ratio (NLR) and platelet-to-lymphocyte ratio (PLR) have been shown to correlate with disease activity in bronchiectasis, with NLR appearing as a relatively classical inflammatory marker for disease activity in bronchiectasis; however, the NLR appears to be a more reliable indicator of systemic inflammation [9].

Further indices, such as the Systemic Immune–Inflammatory Index (SII), which integrates neutrophil, lymphocyte, and platelet counts, have been shown to have prognostic value in bronchiectasis. Elevated SII levels have been associated with increased risk of hospitalization, disease severity, and readmission, highlighting its potential role as a clinically useful biomarker [10,11].

In addition to inflammation, serum albumin—an acute-phase reactant—has been evaluated as an important marker in chronic respiratory diseases, including bronchiectasis. Composite indices that incorporate both inflammatory and nutritional parameters, such as the C-reactive protein–albumin ratio (CAR), have shown significant associations with exacerbation frequency and disease severity in bronchiectasis. Higher CAR levels have been identified as independent predictors of bronchiectasis exacerbations in patients [8].

The hemoglobin–albumin–lymphocyte–platelet (HALP) score is another novel biomarker reflecting both systemic inflammation and acute-phase response. However, its prognostic value has been demonstrated in various inflammatory and pulmonary conditions, including interstitial lung diseases and cardiovascular disorders; its role in bronchiectasis remains largely unclear [12,13].

Furthermore, composite indices such as the Pan-Immune-Inflammation Index (PIV) have been proposed to more comprehensively assess the immune and inflammatory status. Despite increasing interest in these indices, their clinical significance in bronchiectasis, particularly for distinguishing stable disease from exacerbations, remains inadequately investigated.

Although previous studies have evaluated individual inflammatory biomarkers in bronchiectasis, there is a clear lack of studies concurrently assessing multiple composite inflammatory indices in relation to disease activity. As a result, in this study, we aimed to comprehensively evaluate multiple systemic inflammatory markers, including the HALP score, CAR, modified Glasgow Prognostic Score (mGPS), SII, and PIV, between patients with bronchiectasis exacerbation and stable bronchiectasis and to determine their independent predictive value and potential utility as practical biomarkers for clinical risk stratification.

## 2. Materials and Methods

### 2.1. Study Design and Population

**Figure 1:**
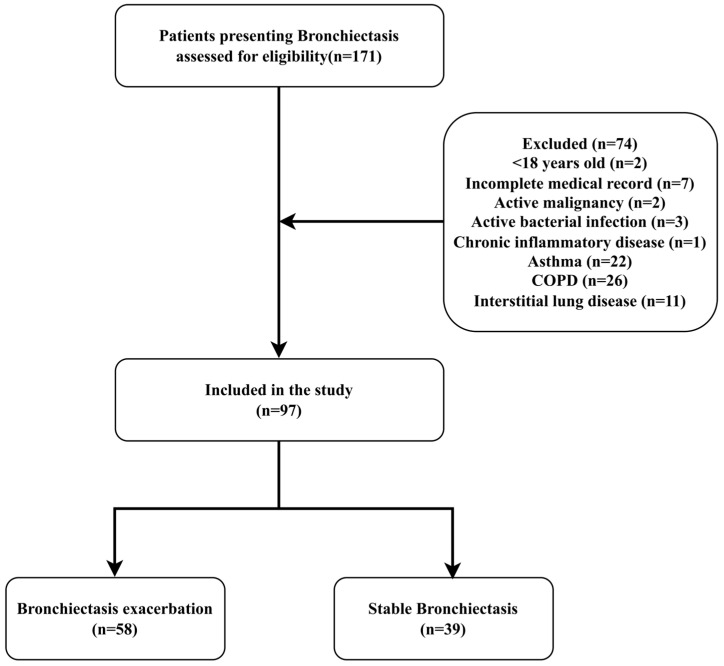
Flow diagram of patient selection and classification of bronchiectasis subtypes.

This retrospective study was conducted in accordance with the principles of the Declaration of Helsinki and with approval from Giresun Training and Research Hospital’s Scientific Research Ethics Committee (decision number 18.02.2026/11). A total of 112 patients diagnosed with bronchiectasis between January 2023 and April 2025 at Giresun Training and Research Hospital were screened, among which 97 were ultimately eligible for inclusion. The diagnosis was established based on clinical presentation and confirmed by high-resolution computed tomography, which demonstrated permanent bronchial dilatation. We included patients in our study who had bronchiectasis recorded in the system based on HRCT findings and who did not have additional lung diseases. They were categorized into stable and exacerbation groups based on their clinical status at the time of evaluation. Exacerbation was defined as a worsening of respiratory symptoms, including increased cough, sputum production, or dyspnea, requiring antibiotic treatment, in accordance with the European Respiratory Society guidelines [14]. Frequent exacerbation was defined as the occurrence of two or more exacerbations within the previous 12 months. Patients with active bacterial infection (n = 3), asthma (n = 22), chronic obstructive pulmonary disease (n = 26), interstitial lung disease (n = 11), malignancy (n = 2), other systemic inflammatory conditions (n = 1), those with incomplete clinical or laboratory data (n = 7), and under 18 years old (n = 2) were excluded (Figure 1).

### 2.2. Data Collection

Demographic and clinical data were collected from medical records, including age, sex, smoking status, comorbidities, disease duration, and exacerbation frequency within the previous 12 months, and information regarding hospitalization history, antibiotic use, and systemic steroid therapy was also recorded. Clinical symptoms such as cough, sputum production, dyspnea, and hemoptysis were evaluated, and radiological involvement was assessed using high-resolution computed tomography (HRCT), which was available for all patients. The presence of *Pseudomonas aeruginosa* colonization was determined from sputum culture results.

### 2.3. Laboratory Measurements and Inflammatory Indices

Laboratory parameters were obtained from routine blood tests, including complete blood count and biochemical markers, and neutrophil, lymphocyte, platelet, hemoglobin, C-reactive protein, albumin, and erythrocyte sedimentation rate values were recorded. Platelet counts are expressed as ×10^3^/µL, whereas other hematological parameters are recorded as absolute values per µL. Several composite inflammatory indices were derived from these parameters, including the neutrophil-to-lymphocyte ratio (NLR), platelet-to-lymphocyte ratio (PLR), Systemic Immune-Inflammation Index (SII: neutrophil × platelet/lymphocyte), Pan-Immune-Inflammation Value (PIV: neutrophil × platelet × monocyte/lymphocyte), C-reactive protein-to-albumin ratio (CAR), C-reactive protein-to-platelet ratio (CPR), HALP (hemoglobin × albumin × lymphocyte/platelet) score, and sedimentation-to-albumin ratio. The modified Glasgow Prognostic Score (mGPS) was calculated using serum C-reactive protein and albumin levels as follows: score 0 (CRP ≤10 mg/L), score 1 (CRP >10 mg/L and albumin >35 g/L), and score 2 (CRP >10 mg/L and albumin <35 g/L). All indices were calculated using laboratory values obtained at the time of clinical evaluation, either at admission for patients with exacerbation or during routine evaluation for stable patients.

### 2.4. Statistical Analysis

All statistical analyses were performed using IBM SPSS Statistics (version 21.0, IBM Corp., Armonk, NY, USA), and continuous variables were assessed for normality using visual methods (histograms and Q–Q plots) and the Shapiro–Wilk test. Since most variables did not follow a normal distribution, continuous variables are reported as medians and interquartile ranges (IQRs), whereas categorical variables are reported as frequencies and percentages.

Comparisons between the stable bronchiectasis group and the exacerbation group were performed using the Mann–Whitney U test for continuous variables and the chi-square test for categorical variables.

Correlation analyses were conducted to evaluate the relationship between inflammatory biomarkers and disease severity. Because hospitalization duration was only applicable to patients with exacerbations, correlation analyses were restricted to patients in the exacerbation group. Associations between variables were evaluated using Spearman’s rank correlation coefficient.

Multicollinearity among inflammatory markers was assessed using the variance inflation factor (VIF).

Multivariate logistic regression analysis was performed to identify independent predictors of the exacerbation phenotype. Separate regression models were constructed for each inflammatory biomarker because of strong correlations among composite inflammatory indices. The models were adjusted for age and bronchiectasis duration, and the results are reported as odds ratios (ORs) with 95% confidence intervals (CIs).

Only selected variables were included in the multivariable model due to the multicollinearity among inflammatory markers. Age and disease duration were considered but not included in the final model, as they were not significant predictors.

The discriminative ability of inflammatory biomarkers for distinguishing stable bronchiectasis from the exacerbation phenotype was assessed using receiver operating characteristic (ROC) curve analysis. The area under the curve (AUC) with 95% confidence intervals was calculated for each biomarker. Optimal cut-off values were determined using the Youden index, and the corresponding sensitivity and specificity are reported.

A two-tailed *p*-value < 0.05 was considered statistically significant.

## 3. Results

A total of 97 patients with bronchiectasis were included in the study; 39 were classified as stable and 58 as experiencing exacerbations of bronchiectasis. The demographic and clinical characteristics of the participants are presented in Table 1.

Patients with bronchiectasis exacerbations had a longer disease duration compared to the stable group (9.21 ± 5.19 vs. 6.69 ± 4.85 years, *p* = 0.018) and also indicated a higher frequency of hospitalizations, antibiotic use, and systemic steroid treatment in the previous year (all *p* < 0.001). Respiratory symptoms such as cough, sputum production, and shortness of breath were significantly more common in the exacerbation group (*p* = 0.003, <0.001, and <0.001, respectively). Radiological involvement was classified as uni- or bilateral, and the extent of bronchiectasis was also assessed according to the number of lobes affected, as shown in Table 1. Bilateral radiological involvement and *Pseudomonas* colonization were also more frequent in patients with exacerbations (*p* < 0.001 and *p* = 0.046, respectively). mGPS scores were significantly higher in patients experiencing exacerbations (*p* < 0.001).

Significant differences in several inflammatory markers were observed between the stable and exacerbation groups. Patients experiencing exacerbation showed higher levels of systemic inflammatory biomarkers, including NLR, PLR, PIV, SII, and CAR (all *p* < 0.05). Conversely, HALP scores were significantly lower in the exacerbation group, suggesting a negative relationship with disease activity (Table 2).

Since hospital stay duration was not applicable to stable patients, correlation analyses were performed among patients experiencing exacerbations, showing that several inflammatory indices were significantly associated with disease severity. Specifically, hospital stay duration showed strong positive correlations with CAR (r = 0.717, *p* < 0.001), CPR (r = 0.583, *p* < 0.001), and sedimentation/albumin ratio (r = 0.593, *p* < 0.001). HALP showed a moderately negative correlation with hospital stay duration (r = −0.580, *p* < 0.001), suggesting that lower HALP values were associated with longer hospitalization duration (Table 3).

Univariate logistic regression analysis was performed to evaluate the relationship between clinical and inflammatory variables and the exacerbation phenotype (Table 4).

The analysis revealed that HALP, NLR, SII, CAR, mGPS, hospitalization history, and bilateral radiological involvement were significantly associated with the exacerbation phenotype. *Pseudomonas* colonization showed borderline significance.

Low HALP levels were inversely associated with the probability of exacerbation (OR = 0.637, *p* < 0.001). Similarly, increased NLR (OR = 1.326, *p* = 0.010) and SII (OR = 1.001, *p* = 0.008) values were positively associated with the exacerbation phenotype. However, clinical variables were examined, and hospitalization history (OR = 0.028, *p* = 0.001) and bilateral radiological involvement (OR = 0.158, *p* < 0.001) were found to be strong predictors compared to inflammatory markers. This finding suggests that clinical factors reflecting disease burden may be a better determinant of the exacerbation phenotype. Although CAR and mGPS variables were found to be significantly associated with exacerbation, odds ratios and confidence intervals for these variables could not be reliably calculated due to quasi-complete separation. Since this indicates a strong but statistically unstable association, these variables were excluded from the multivariate analysis.

A high level of multicollinearity (VIF >10) was found among inflammatory markers; therefore, representative variables selected based on both statistical criteria and clinical significance were included in the multivariate model. The number of lobes involved was evaluated but not included in the multivariate model due to its linear relationship with radiological involvement.

Multivariate logistic regression analysis was performed using variables that were found to be significant in the univariate analysis and considered clinically important (Table 5).

The analysis revealed that history of hospitalization (OR = 0.042, *p* = 0.004) and bilateral radiological involvement (OR = 0.188, *p* = 0.002) were independent predictors of the exacerbation phenotype. The HALP level showed borderline significance in the adjusted model (OR = 0.749, *p* = 0.054). The reduced effect of inflammatory markers in the multivariate model suggests that their association with exacerbation may be partly related to the underlying disease severity. Due to the lack of validated composite disease severity scores, clinical variables such as hospitalization history and radiological extent were explicitly included in the regression models as indirect indicators of disease severity.

The established model was found to have a good fit (Hosmer–Lemeshow *p* = 0.901) and moderate explanatory power (Nagelkerke R^2^ = 0.504).

**Figure 2:**
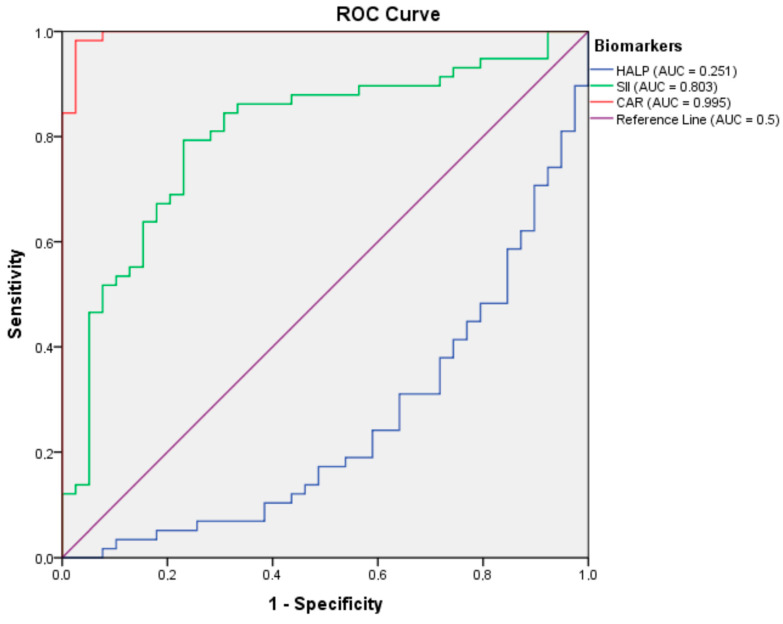
Receiver operating characteristic (ROC) curves of inflammatory biomarkers for predicting the exacerbation phenotype in bronchiectasis. CAR demonstrated the highest discriminative ability, followed by SII, whereas HALP showed an inverse association.

Receiver operating characteristic (ROC) curve analysis was performed to evaluate the ability of inflammatory markers to differentiate stable bronchiectasis from the exacerbation phenotype, with the results shown in Table 6 and Figure 2.

Among the biomarkers evaluated, CAR showed the highest discriminatory performance, although this very high AUC value may reflect potential overfitting given the sample size, with an area under the curve (AUC) of 0.995 (95% CI: 0.986–1.000, *p* < 0.001). A CAR cut-off value of 0.21 provided a sensitivity of 84.5% and a specificity of 97.4% for identifying patients with exacerbation.

SII also showed good discriminatory ability (AUC = 0.803), followed by NLR (AUC = 0.789) and PIV (AUC = 0.766). HALP showed an inverse relationship with exacerbation status (AUC = 0.251), reflecting an inverse association, with lower HALP values observed in patients with exacerbation.

## 4. Discussion

In this study, we comprehensively evaluated multiple systemic inflammatory indices in patients with bronchiectasis and demonstrated that these biomarkers were significantly different between stable and exacerbation phenotypes. We found that several systemic inflammatory indices, including CAR, SII, NLR, and PIV, were significantly elevated in patients with exacerbations, while HALP was significantly reduced, with HALP and SII emerging as independent predictors of exacerbation status and CAR demonstrating the highest discriminative performance among the evaluated markers. These findings highlight the potential role of composite inflammatory indices as practical tools for evaluating disease activity in bronchiectasis.

Bronchiectasis is driven by a self-perpetuating “vicious cycle” of chronic infection, impaired mucociliary clearance, and persistent inflammation, while composite indices such as NLR, PLR, and SII reflect the balance between innate and adaptive immunity and are associated with disease severity and prognosis [5]. These markers are particularly valuable due to their accessibility and cost-effectiveness, making them attractive for routine clinical use. Systemic inflammation is increasingly recognized as a key component of disease activity, particularly during exacerbations [7]. Our findings support this pathophysiological framework, as elevated composite inflammatory indices were observed in patients with exacerbations.

SII is attracting increasing attention as a comprehensive marker reflecting the systemic immune response. Previous studies have shown that high SII levels are associated with an increased risk of hospitalization, disease severity, and re-hospitalization in patients with bronchiectasis [10,11]. In light of these findings, we observed that SII was significantly higher in patients experiencing exacerbations and remained an independent predictor of the exacerbation phenotype. Although the odds ratio for SII appears numerically small, this is most likely due to the large scale of the variable, a phenomenon frequently observed in composite hematological indices. Future analyses using standardized values (e.g., per standard deviation increase) may provide more clinically interpretable effect estimates.

One of the most remarkable findings of this study is the role of the HALP score, which was significantly lower in patients with exacerbations and demonstrated an inverse relationship with disease severity, as reflected by its negative correlation with hospitalization duration. Moreover, HALP showed borderline significance as an independent predictor in the adjusted model. These findings suggest that HALP may reflect an inverse association with disease activity rather than a direct protective effect. Although HALP has been investigated in various inflammatory and pulmonary diseases, including interstitial lung diseases and cardiovascular conditions, its role in bronchiectasis is not well-established [12,13,15]. Therefore, our study provides novel evidence supporting the potential value of HALP as a biomarker in bronchiectasis, but given the cross-sectional design, causal inferences cannot be made.

Previous studies have demonstrated that lower HALP scores are associated with worse survival outcomes and more aggressive disease characteristics in patients with malignancies [16]. Moreover, recent systematic reviews and meta-analyses have confirmed that low HALP is consistently associated with poor prognosis and adverse clinical outcomes, further emphasizing its potential clinical utility [17]. The biological rationale for HALP rests on its ability to integrate multiple dimensions of patient status, including anemia, malnutrition, immune competence, and systemic inflammation [18]. In this context, our findings suggest that decreased HALP may reflect impaired immuno-nutritional status in patients with bronchiectasis exacerbations, potentially contributing to increased disease activity and vulnerability to exacerbations.

Among these biomarkers, CAR demonstrated high discriminatory performance in our study. CAR, integrating CRP and albumin levels, is a strong composite biomarker of acute-phase response and systemic inflammation and has been consistently identified as an independent predictor of increased mortality and adverse clinical outcomes across diverse conditions, including sepsis and malignancies [19,20]. However, it should be noted that albumin is not a specific marker of nutritional status and can be affected by various factors, including systemic inflammation, infection, and comorbidities. Therefore, low albumin levels do not necessarily indicate malnutrition, especially in advanced clinical settings where inflammatory processes play a more prominent role. In our study, CAR demonstrated the strongest discriminatory performance with a very high AUC of 0.995, but this finding should be interpreted cautiously due to potential overfitting and the possibility of shared components with clinical assessment. This finding is broadly consistent with previous research showing that CAR reflects both inflammatory load and nutritional status and is associated with exacerbation frequency in bronchiectasis [8]. However, the relatively small sample size and single-center design may have contributed to an overestimation of the predictive performance, and further validation in larger, multicenter cohorts is needed.

In addition, several studies in other disease settings have demonstrated that elevated CAR is consistently associated with worse overall survival, disease progression, and increased mortality across different cancer populations, highlighting its potency as a prognostic marker [21]. Furthermore, CAR has been shown to be better or complementary to other inflammation-based scores, including NLR, PLR and mGPS, in predicting patient outcomes [22]. These findings are further supported by studies on nasopharyngeal carcinoma, in which CAR has been shown to be an independent predictor of survival and disease progression [20,23,24].

Inflammation-based scoring systems, such as the Modified Glasgow Prognostic Score (mGPS), have been widely validated as reliable prognostic indicators in a variety of clinical settings. Derived from C-reactive protein and albumin levels, the mGPS primarily reflects both systemic inflammatory load and nutritional status and has been consistently associated with survival outcomes in different patient populations [25,26]. Albumin levels are affected by numerous factors, including inflammation and comorbidities, and should therefore not be interpreted as a specific indicator of nutritional status on their own. These findings highlight the value of integrating multiple parameters into a single index rather than relying on isolated biomarkers. In the context of our study, this concept supports the use of inflammation-based composite markers in bronchiectasis and suggests that such indices can provide valuable information about disease activity and patient stratification. However, mGPS was not included in the final multivariable model due to model instability, which may warrant future research.

Consistent with previous studies, NLR appeared to be a more reliable marker of systemic inflammation than PLR, showing stronger associations with disease activity [9]. In addition, PIV was significantly elevated in patients with exacerbations, suggesting that broader immune–inflammatory interactions may contribute to disease activity. However, neither NLR nor PIV retained independent predictive value in multivariable analysis, which may reflect overlapping inflammatory pathways or potential collinearity among composite indices. Previous studies have also emphasized the biological heterogeneity of bronchiectasis and the limited ability of single biomarkers to fully capture disease complexity, highlighting the value of integrating multiple clinical and inflammatory parameters [3]. In this context, our findings support the use of composite indices to provide a more comprehensive assessment of disease activity and potential clinical utility in bronchiectasis.

In a recent prospective cohort study, Chen et al. demonstrated that a substantial proportion of bronchiectasis patients exhibit at least one positive type 2 biomarker, including blood eosinophils, FeNO, or total IgE, suggesting the coexistence of multiple inflammatory pathways within the same disease spectrum. However, their findings also indicated that traditional systemic inflammatory markers, particularly NLR and microbial factors such as *Pseudomonas aeruginosa* colonization, remain stronger predictors of exacerbation risk compared to type 2 biomarkers [27]. These findings are in line with our results, where systemic inflammation-based indices such as SII, CAR, and NLR demonstrated strong associations with exacerbation status, supporting the central role of neutrophil-driven inflammation in bronchiectasis. At the same time, the observed inverse relationship of HALP with exacerbation suggests that composite indices incorporating nutritional and hematological parameters may capture additional dimensions of disease activity beyond pure inflammatory burden. These findings also support the heterogeneity of inflammatory endotypes in bronchiectasis.

From a clinical perspective, the biomarkers evaluated in this study are inexpensive, readily available, and easily derived from routine laboratory tests, making them practical for daily use. Our findings support the concept that bronchiectasis should be assessed using a multi-dimensional biomarker approach rather than a single parameter. In this context, indices such as HALP, SII, and CAR may assist clinicians in risk stratification by identifying patients at higher risk of exacerbations, enabling closer monitoring and more timely interventions. In clinical practice, patients with elevated inflammatory indices may benefit from closer follow-up, optimization of medical therapy, or early initiation of treatment during worsening symptoms. Conversely, lower-risk patients may be managed with standard follow-up strategies. Overall, these markers may contribute to individualized risk stratification and support clinical decision-making in routine practice.

However, several limitations should be acknowledged. The retrospective and single-center design may have introduced potential selection bias, and the relatively small sample size may have influenced the observed predictive performance of certain biomarkers. Furthermore, data deficiencies inherent in the nature of retrospective design, such as the inconsistent availability of pulmonary function test data, may have prevented the calculation of validated composite severity scores like FACED and BSI, thus limiting a comprehensive assessment of disease severity. To partially address this limitation, clinically relevant surrogate markers, including hospitalization history and radiological extent, were incorporated into the analysis. In this context, radiological involvement was evaluated not only as uni- or bilateral disease but also based on the number of affected lobes, allowing for a more detailed assessment of disease burden. Previous studies have demonstrated that clinical variables such as exacerbation frequency, radiological extent, and inflammatory burden are closely associated with disease severity in bronchiectasis [28,29]. Therefore, in retrospective settings where comprehensive severity scores are not always available, such parameters may serve as pragmatic indicators of disease burden. In our study, the identification of these variables as significant determinants in multivariate analysis further supports the role of disease severity in shaping the exacerbation phenotype. Moreover, the loss of significance of certain inflammatory markers in the adjusted models suggests that their effects may be, at least in part, mediated through disease severity rather than acting as independent determinants. The lack of external validation also limits the generalizability of these findings, and causal relationships cannot be established due to the observational nature of the study. Therefore, further large-scale, prospective, multicenter studies are needed to confirm these results and clarify their clinical applicability.

## 5. Conclusions

In conclusion, systemic inflammatory indices acquired from routine hematological parameters are strongly associated with the exacerbation phenotype in bronchiectasis. Among these, CAR demonstrated a highly discriminative ability, SII emerged as an independent predictor, and HALP showed borderline significance as a predictor of exacerbation status. In particular, the inverse association of HALP with disease activity suggests that combined composite inflammatory indices may provide additional insight beyond traditional markers. These findings support the potential role of simple, cost-effective biomarkers in the clinical assessment and risk stratification of bronchiectasis patients.

## Figures and Tables

**Table 1:** Demographic and clinical characteristics of patients with stable bronchiectasis and bronchiectasis exacerbations (n: 97).

Variables	Stable Bronchiectasis (n: 39)	Bronchiectasis Exacerbations (n: 58)	*p*-Value
Age, years (mean ± SD)	54.23 ± 17.52	60.69 ± 16.36	0.067
BMI	29.2 ± 6.18	27.9 ± 6.19	0.639
Sex, n (%)			
Female	22 (56.4)	26 (54.2)	0.302
Male	17 (43.6)	32 (55.2)	
Smoking status, n (%)			0.136
Current smoker	28 (46.7)	32 (53.3)	
Former smoker	10 (34.5)	19 (65.5)	
Never	1 (12.5)	7 (12.1)	
Etiology of bronchiectasis			
İdiopathic	27 (69.2)	26 (44.8)	0.021
Tuberculosis	10 (25.6)	24 (41.4)	
Post-infectious	0 (0)	7 (12.1)	
Others	2 (5.1)	1 (1.7)	
Bronchiectasis duration, years (mean ± SD)	6.69 ± 4.85	9.21 ± 5.19	0.018
Bronchiectasis exacerbations in the past 12 months			
None	29 (74.4)	20 (40.8)	<0.001
Yes	10 (25.6)	38 (65.5)	
Bronchiectasis exacerbations requiring hospitalization in the last 12 months			
No	37 (94.9)	32 (55.2)	<0.001
Yes	2 (5.1)	26 (44.8)	
Symptoms (yes, n, %)			
Cough	29 (74.4)	56 (96.6)	0.003
Sputum	17 (43.6)	49 (84.5)	<0.001
Dyspnea	11 (28.2)	42 (72.4)	<0.001
Hemoptysis	3 (7.7)	4 (6.9)	0.589
Having received antibiotic treatment for bronchiectasis within the last 12 months			
Zero	26 (74.4)	10 (17.2)	<0.001
One	9 (23.1)	24 (41.4)	
Twice or more	1 (2.6)	24 (41.4)	
Having received systemic steroid treatment for bronchiectasis within the last 12 months			
None	38 (97.4)	36 (62.1)	<0.001
Yes	1 (2.6)	22 (37.9)	
Any comorbidities (yes, n, %)	13 (33.3)	30 (51.7)	0.072
Hypertension, n (%)	12 (30.8)	30 (51.7)	0.060
Diabetes mellitus type 2, n (%)	1 (2.6)	5 (8.6)	0.396
Coronary artery diseases, n (%)	8 (20.5)	9 (15.5)	0.591
mGPS			
0	39 (100)	9 (15.5)	<0.001
1	0 (0)	34 (58.6)	
2	0 (0)	15 (25.9)	
mMRC			
<2	36 (52.9)	32 (55.2)	<0.001
>2	3 (7.7)	26 (44.8)	
Radiological extent of bronchiectasis, n (%)			
Unilateral	30 (76.9)	20 (20)	<0.001
Bilateral	9 (23.1)	38 (65.5)	
Lobar involvement, n (%)			
Left upper lobe	4 (10.3)	20 (34.5)	0.008
Lingula	9 (23.1)	17 (29.3)	0.641
Left lower lobe	22 (56.4)	35 (60.3)	0.834
Right upper lobe	3 (7.7)	13 (22.4)	0.092
Right middle lobe	8 (20.5)	19 (32.8)	0.249
Right lower lobe	19 (48.7)	36 (62.1)	0.215
*Pseudomonas aeruginosa* colonization			
No	38 (97.4)	48 (82.8)	0.046
Yes	1 (2.6)	10 (17.2)	

Variables with a normal distribution are presented as mean ± standard deviation, while non-normally distributed or ordinal variables are presented as median (25th–75th percentile). Categorical variables are expressed as counts and percentages. Group comparisons were performed using Student’s *t*-test or the Mann–Whitney test for continuous variables and the chi-square test for categorical variables, as appropriate. Abbreviations: mGPS: modified Glasgow Prognostic Score. Footnote: Although group classification was based on exacerbations within the past 6 months, exacerbation history over the prior 12 months is also reported as a descriptive variable.

**Table 2:** Comparison of inflammatory biomarkers between stable bronchiectasis and exacerbation groups.

Variable	Stable Bronchiectasis (n = 39) Median (IQR)	Bronchiectasis Exacerbation (n = 58) Median (IQR)	*p*-Value
HALP	4.82 (3.50–6.01)	2.90 (1.41–4.24)	<0.001
NLR	1.96 (1.32–2.75)	3.92 (2.56–6.99)	<0.001
PLR	119.35 (96.60–156.67)	165.39 (133.86–266.39)	<0.001
PIV	279.28 (183.81–515.43)	894.14 (361.86–1885.72)	<0.001
SII	567.00 (346.06–774.92)	1433.18 (790.91–2565.61)	<0.001
CAR	0.05 (0.02–0.11)	1.18 (0.30–3.15)	<0.001

Data are presented as medians (interquartile range, IQR). Group comparisons were performed using the Mann–Whitney U test. Abbreviations: HALP: hemoglobin, albumin, lymphocyte, platelet score; NLR: neutrophil-to-lymphocyte ratio; PLR: platelet-to-lymphocyte ratio; PIV: Pan-Immune-Inflammation Value; SII: Systemic Immune-Inflammation Index; CAR: C-reactive protein-to-albumin ratio; IQR: interquartile range.

**Table 3:** Correlation between hospitalization duration and inflammatory biomarkers in patients with exacerbation (n = 58).

Variable	Correlation Coefficient (r)	*p*-Value
CAR	0.717	<0.001
CPR	0.583	<0.001
Sedimentation/Albumin ratio	0.593	<0.001
HALP	−0.580	<0.001

Abbreviations: CAR: C-reactive protein-to-albumin ratio; CPR: C-reactive protein-to-platelet ratio; HALP: hemoglobin, albumin, lymphocyte, platelet score. Note: Correlations were calculated using Spearman correlation analysis among patients with exacerbation. Positive values indicate a direct association with hospitalization duration, whereas negative values indicate an inverse association.

**Table 4:** Univariable logistic regression analysis of factors associated with the exacerbation phenotype (dependent variable: exacerbation status).

Variable	OR	95% CI	*p*-Value
HALP	0.637	0.502–0.808	<0.001
NLR	1.326	1.070–1.643	0.010
SII	1.001	1.000–1.001	0.008
CAR	–	–	0.002
mGPS	–	–	<0.001
History of hospitalization	0.028	0.004–0.219	0.001
Bilateral radiological involvement	0.158	0.063–0.397	<0.001
Pseudomonas colonization	0.126	0.015–1.031	0.053

Abbreviations: OR: odds ratio; CI: confidence interval; CAR: C-reactive protein-to-albumin ratio; mGPS: modified Glasgow Prognostic Score. For CAR and mGPS, odds ratios and 95% confidence intervals could not be reliably estimated due to quasi-complete separation, leading to model instability.

**Table 5:** Multivariate logistic regression models identifying predictors of the exacerbation phenotype.

Model A-Dependent: Exacerbation Phenotype			
Variable	OR	95% CI	*p*-Value
HALP	0.749	0.558–1.005	0.054
History of hospitalization	0.042	0.005–0.356	0.004
Bilateral radiological involvement	0.188	0.065–0.542	0.002

Multivariate logistic regression analysis was performed to identify independent predictors of the exacerbation phenotype in bronchiectasis. The models were adjusted for hospitalization (yes vs. no) and bilateral radiological involvement. Odds ratios (ORs) are presented with 95% confidence intervals (CIs). The model demonstrated good fit (Hosmer–Lemeshow *p* = 0.901) and moderate explanatory power (Nagelkerke R^2^ = 0.504). Abbreviations: OR: odds ratio; CI: confidence interval; HALP: hemoglobin, albumin, lymphocyte, platelet score.

**Table 6:** ROC analysis of inflammatory biomarkers.

Marker	AUC	95% CI	*p*	Cut-Off	Sensitivity	Specificity
CAR	0.995	0.986–1.000	<0.001	0.21	84.5%	97.4%
SII	0.803	0.712–0.894	<0.001	782	79.3%	76.9%
NLR	0.789	0.697–0.881	<0.001	3.0	65.5%	76.9%
PIV	0.766	0.670–0.861	<0.001	471	72.4%	66.7%
HALP *	0.251 *	0.152–0.350	<0.001	≤2.6	62.1%	87.2%

* HALP showed an inverse association (AUC = 0.251), indicating lower HALP values in the exacerbation group. Abbreviations: AUC: area under curve; CAR: C-reactive protein-to-albumin ratio; SII: Systemic Immune-Inflammation Index; NLR: neutrophil-to-lymphocyte ratio; PIV: Pan-Immune-Inflammation Value; HALP: hemoglobin, albumin, lymphocyte, platelet score. Sensitivity and specificity values were calculated based on the optimal cut-off values determined by ROC analysis.

## Data Availability

The datasets used and/or analyzed during the current study are available from the corresponding author on reasonable request.

## Abbreviations

The following abbreviations are used in this manuscript:

NLR (Neutrophil-to-Lymphocyte Ratio)	neutrophil count/lymphocyte count
PLR (Platelet-to-Lymphocyte Ratio)	platelet count/lymphocyte count
SII (Systemic Immune-Inflammation Index)	neutrophil × platelet/lymphocyte
PIV (Pan-Immune-Inflammation Value)	neutrophil × platelet × monocyte/lymphocyte;
CAR (C-Reactive Protein-to-Albumin Ratio)	CRP/albumin
CPR (C-Reactive Protein-to-Platelet Ratio)	CRP/Platelet
mGPS	modified Glasgow prognostic score
HALP score	hemoglobin × albumin × lymphocyte/platelet
ESR	Erythrocyte sedimentation rate
Sedimentation/Albumin Ratio	ESR/albumin
